# Creativity-Contingent Rewards, Intrinsic Motivation, and Creativity: The Importance of Fair Reward Evaluation Procedures

**DOI:** 10.3389/fpsyg.2020.00974

**Published:** 2020-06-11

**Authors:** Erik Andreas Saether

**Affiliations:** Norwegian University of Science and Technology, Trondheim, Norway

**Keywords:** intrinsic motivation, procedural justice, creativity, rewards, creativity-contingent rewards, divergent thinking, pay for performance

## Abstract

Pay for performance is a common practice used by organizations to increase employees’ motivation and performance, and creativity-contingent rewards have been shown to support creativity. But are all creativity-contingent rewards equal? Procedural justice can potentially affect the way that creativity-contingent rewards impact employees’ intrinsic motivation and creativity. To shed light on this practice-relevant issue, this study investigates how aspects of procedural justice—reward allocation clarity and reward evaluation fairness—impact *changes* in intrinsic motivation and creativity in the presence of creativity-contingent rewards. Using an incomplete factorial pretest–posttest between subjects design with four reward conditions and one control (no reward) condition, I analyzed changes in intrinsic motivation and creativity. Relative to the control condition, significant increases in both intrinsic motivation and creativity were found in the reward conditions with high evaluation fairness. However, reward allocation clarity did not yield any significant effects on changes in intrinsic motivation and creativity. The results highlight the importance of fair evaluation procedures for determining rewards if creativity-contingent rewards are to increase both intrinsic motivation and creativity.

## Introduction

*Creativity*, defined as the production of novel and useful ideas (Amabile, 1996), is often desired by organizations because it contributes to organizational innovation (Amabile, 1988; Anderson et al., 2014), and *intrinsic motivation*—i.e., doing activities out of sheer interest, or enjoyment (Ryan and Deci, 2000)—is necessary for creativity to occur (Amabile, 1996). Pay for performance is a common practice used by organizations to increase their employees’ motivation and performance (Gupta and Shaw, 2014); however, there has been disagreement about the effects of monetary rewards on both motivation and creativity (Gerhart and Fang, 2015). Relatedly, scholars have suggested that new research should study creativity-contingent rewards (performance-contingent rewards dependent on creativity) and their relationship to intrinsic motivation and creativity (Hughes et al., 2018; Fischer et al., 2019).

A meta-analysis by Byron and Khazanchi (2012) found that creativity-contingent rewards can foster creativity because they signal that creativity is valued and direct efforts toward creative performance (Eisenberger and Shanock, 2003). Amabile (1993, 1997) also suggested that there are potentially beneficial synergistic effects between intrinsic and extrinsic motivation in relation to creativity. Recently, the effects of creativity-contingent rewards on creativity have been studied more prevalently (e.g., Malik and Butt, 2017). Some of these studies have also investigated the boundary conditions for these types of rewards (Malik et al., 2015; Yoon et al., 2015); however, *procedural justice*—fairness related to decision-making processes (Leventhal, 1980)—has been largely absent in research on rewards’ effects on intrinsic motivation and creativity. This is surprising considering that procedural justice has been shown to impact affect (Weiss et al., 1999; Krehbiel and Cropanzano, 2000), intrinsic motivation (Zapata-Phelan et al., 2009; Olafsen et al., 2015), multiple performance measures including task performance (Colquitt et al., 2001), and creativity (Simmons, 2011). Therefore, it is logical to assume that procedural justice could affect the relationship between rewards, intrinsic motivation, and creativity.

There are at least two mechanisms for the positive impact of justice on intrinsic motivation and creativity. First, as guided by fairness theory (Folger and Cropanzano, 1998), procedural justice may lead to intrinsic motivation and performance through positive affect (Zapata-Phelan et al., 2009). Since intrinsic motivation is commonly associated with enjoyment, pleasure, and positive affect (Pretty and Seligman, 1984; Vallerand, 1997; Deci et al., 1999; Silvia, 2008), then justice’s causation of positive affect will consequently increase intrinsic motivation and creativity.

Secondly, self-determination theory (SDT; Deci and Ryan, 1985) helps to explain how fair procedures can influence intrinsic motivation and performance on creative tasks. The multiple needs model of justice (Williams, 1997) proposed that fair treatment fulfills fundamental human needs, including control, positive self-regard, and belonging (Cropanzano et al., 2001). These needs are conceptually similar to SDT’s basic needs of autonomy, competence, and relatedness, which when fulfilled, will lead to intrinsic motivation. According to SDT, motivation quality is more important than quantity, and the highest quality of motivation is intrinsic (Ryan and Deci, 2000). Intrinsic motivation is the most autonomous and self-determined form of motivation, and self-determined forms of motivation are critical in relation to complex tasks (Gagné and Deci, 2005). Intrinsic motivation is necessary for creativity (Amabile, 1996), since it may increase novelty (Zhou, 1998), persistence (Oldham and Cummings, 1996), and flexibility (Amabile, 1996). Thus, as Gagné and Forest (2008) and Weibel et al. (2014) suggested, procedural justice in relation to monetary rewards may increase intrinsic motivation and performance, such as creativity.

This study aimed to provide a better understanding of the effects of creativity-contingent monetary rewards under conditions of procedural justice on changes in individuals’ intrinsic motivation and creativity by using a behavioral experiment. Studies on creativity often focus on output measures of creativity, but this study looks at changes in creativity with a controlled pretest–posttest design to isolate causal factors in the reward conditions. In an attempt to make the study practically relevant to employees in organizations, adult participants working for monetary gain were used. This is important because many prior laboratory studies which investigated extrinsic rewards, intrinsic motivation, and creativity have used students who have not been initially offered rewards to participate (Lepper et al., 1973; Amabile et al., 1986; Selart et al., 2008). Furthermore, the procedural justice conditions were inspired by interviews with practitioners, including R&D and patent professionals. These interviews illuminated allocation clarity and evaluation fairness as two elements of procedural justice that could influence the impact of creativity-contingent monetary rewards on intrinsic motivation and creativity.

Specifically, by employing a behavioral experiment, I investigated whether creativity-contingent rewards under procedural justice conditions of *reward evaluation fairness* (the fairness of the evaluation process used to determine rewards) and *reward allocation clarity* (the clarity of the reward allocation process) can cause changes in intrinsic motivation and creativity. I predicted that the participants in conditions of high reward evaluation fairness and conditions of high reward allocation clarity would exhibit increases in both intrinsic motivation and creativity. Moreover, although Simmons (2011) demonstrated a direct effect between procedural justice and creativity, I expected that intrinsic motivation would mediate the relationships between reward evaluation fairness and creativity and between reward allocation clarity and creativity.

## Methods

### Experiment Participants and Design

**FIGURE 1:**
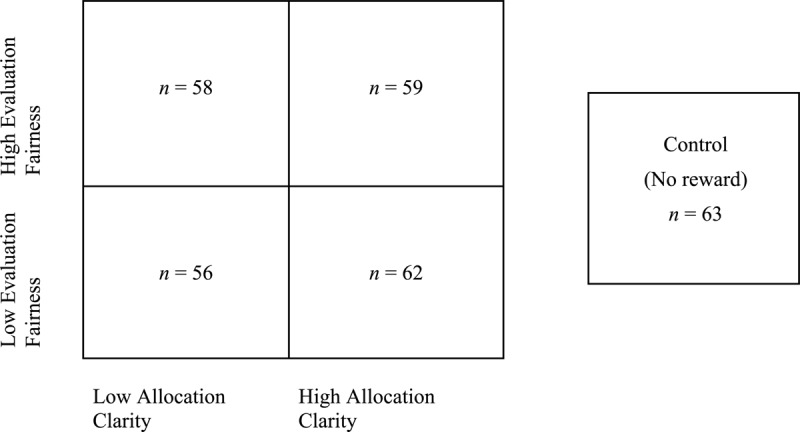
Experiment design—five conditions.

The experiment used an incomplete factorial pretest–posttest between subjects design with a total of five conditions; see Figure 1 for a visual representation. Participants were randomly assigned to one of the five conditions: a control (no reward) condition and a reward group separated into four unique conditions in a 2 (low allocation clarity versus high allocation clarity) × 2 (low evaluation fairness versus high evaluation fairness) matrix.

Three hundred five participants were initially recruited from Amazon’s Mechanical Turk (MTurk), an online crowdsourcing platform that offers subjects who are sufficiently diverse and representative of non-college populations and provides results that meet or exceed psychometric standards associated with published research (Buhrmester et al., 2011). Participants only qualified for the study if they had approval ratings above 98% for previous MTurk tasks and were in the United States (US), Canada, or the United Kingdom (UK). Participants from these countries were chosen since their national cultures are highly individualistic with small power distances (Hofstede, 1980), and they were assumed to behave similarly in regard to rewards. After collecting the data, it was detected that five participants had IP addresses outside of the US, Canada, and the UK, while two additional participants had repeat responses. These responses were subsequently removed, leaving a total of 298 responses for the analysis. Each condition had a similar number of participants (minimum = 56, maximum = 63), while 85.6% of participants resided in the US, 9.4% lived in Canada, and 5% were in the UK. The average age range of participants was 26–34 years, 49% were female, and 68% had completed some form of higher education.

### Procedure

Participants were given a link to the experiment website, and all participants were informed that for participating in an academic study on creativity, they would receive $0.80 (USD) upon completion. This ensured that participants were already motivated by monetary gain. The experiment website included a total of nine pages containing instructions, survey questions, and creativity tasks. On the first page, participants were given the following introduction: “You will be asked to do two short activities and respond to some multiple response items. It should take a total of 6–8 min. There are no ‘Back’ buttons, so read the directions carefully before clicking the ‘Next’ button. Both activities are of a similar nature. You will be provided the name of an object and then will be asked to write down as many uses as possible for that object in 2 min. Try to be creative!”

Then, participants were directed to the next page where they did the first task, establishing a pretest measure of their creativity. Specifically, they were asked to write as many creative uses as they could for a brick in 2 min, a divergent thinking exercise known as the alternative uses task (AUT; Guilford, 1967). After completing the brick AUT, all participants responded to four questions to establish a pretest measure of their intrinsic motivation. Then, all participants were informed that they would do a similar activity on the next page. It was at this point that the manipulations were enacted.

#### Reward Manipulation

Participants were randomly assigned to one of the five conditions before doing the next AUT. Participants in each of the four reward conditions were told: “A $20 (USD) reward will be given to the top 5 participants with the highest number of creative uses for the next activity.”

Since explicit instructions to be creative can increase divergent thinking and creativity (Harrington, 1975; O’Hara and Sternberg, 2001), it was important to provide the control condition with a creativity directive. This was an essential clue that creativity was desired, thereby contrasting the reward conditions and control condition on the presence or absence of a monetary reward, and not the informational, or goal-setting value of a creativity objective. Thus, in the control condition, participants were given the following message: “Important! On the next activity, try to provide as many creative uses as possible.”

#### Reward Allocation Clarity Manipulation

Then, participants in the four reward conditions were given additional information. In the high allocation clarity condition, participants were informed: “Important! When: The reward will be given in exactly two weeks from today.” On the other hand, participants in the low allocation clarity condition were told: “Important! When: The reward will be given at an undetermined point in the future.”

#### Reward Evaluation Fairness Manipulation

Furthermore, in the high reward evaluation fairness condition, participants were told: “How: The creativity of ideas will be determined by a scoring process based on the independent analysis of two expert creativity judges.” Conversely, participants in the low reward assessment fairness condition were informed: “How: The creativity of ideas will be determined by a scoring process based on the gut feeling of a university student.”

Next, all participants did their second and final task, an AUT for which they were asked to write down as many creative uses for a coffee mug in 2 min. This established a posttest measure of creativity for all participants. Finally, all participants responded to four additional items to determine their posttest measure of intrinsic motivation. Additionally, participants in the reward conditions responded to manipulation checks to determine whether the manipulations had been effective.

### Measures

*Intrinsic motivation* was measured twice—pretest (α = 0.95) and posttest (α = 0.97)—with the four-item intrinsic motivation subscale of the Situational Motivation Scale (SIMS) developed by Guay et al. (2000). Scale items included: “I thought the activity was interesting,” “I thought the activity was pleasant,” “I thought the activity was fun,” and “I felt good when doing the activity.” All items used a 7-point unipolar agreement response scale ranging from 1 = *Not at all* to 7 = *Agree completely.*

*Creativity* was measured for the brick (pretest) and the coffee mug (posttest) using a variation of the consensual assessment technique (Amabile, 1982), a method of subjective scoring that is appropriate for scoring creativity with divergent thinking tasks (Silvia et al., 2008). Furthermore, an overall creativity score was appropriate in this case, since participants were instructed to be creative (Harrington, 1975; Silvia et al., 2008) and simple averages of participants’ ideas would unnecessarily hurt participants with high fluency scores. Two independent raters with experience assessing creative ideas (a retired school teacher and an advertising agency manager), and blind to both the study predictions and conditions, scored the creativity of participants’ ideas for uses for the brick and coffee mug on a 3-point scale (0 = not at all creative, 1 = somewhat creative, and 2 = very creative). The raters were informed beforehand that creative ideas are those that are both novel and useful. Each idea was scored by each rater, and then these scores were added to provide a sum-total score for each participant. Then, the sum-total scores of each rater (ICC_brick_ = 0.77, ICC_mug_ = 0.81) were averaged for each participant, providing a pretest and a posttest creativity score—one for the brick and one for the coffee mug.

Since the purpose of the study was to observe and test changes in intrinsic motivation and creativity, change scores were ultimately used as dependent variables. Pretest (*M* = 3.73, *SD* = 2.54) and posttest (*M* = 4.31, *SD* = 2.29) creativity scores were highly and significantly correlated (0.52, *p* < 0.001), and the measures were deemed similar enough to use to produce change scores. These were calculated by subtracting the pretest scores from the posttest scores and hereafter referred to as *intrinsic motivation change* and *creativity change*.

### Manipulation Checks

The manipulation checks also used a 7-point response scale ranging from 1 = *Not at all* to 7 = *Agree completely*. To check that *reward allocation clarity* had been adequately manipulated, participants in the reward conditions responded to three items (α = 0.96), including, “It is clear when the $20 rewards will be given.” In addition, the *reward evaluation fairness* manipulation was measured with three items (α = 0.96); a sample item is “The way that my ideas will be evaluated is fair.”

## Results

### Initial Check of Demographics and Alternative Uses Task Experience Between Conditions

Participants were assigned to condition at random, but as an added measure of control, an initial analysis of variance (ANOVA) confirmed that participants in the five conditions did not significantly differ from one another in terms of age, sex, education level, country of inhabitance, or whether participants had done an AUT before.

### Descriptive Statistics

Table 1 presents the means, standard deviations, and Pearson’s correlations among the variables of interest for the analyses. The clarity and fairness scales were only measured in the reward conditions (*n* = 235), while the intrinsic motivation and creativity variables reflect all conditions (*n* = 298).

**TABLE 1:** Means, standard deviations, and pearson correlations.

	***M***	***SD***	**1**	**2**	**3**	**4**	**5**	**6**	**7**	**8**
1. Reward^a^	0.79	0.41	–							
2. Reward evaluation fairness^b^	4.67	1.49	–							
3. Reward allocation clarity^b^	4.52	2.03	–	0.42**						
4. Pretest intrinsic motivation	4.57	1.49	–0.10	0.44**	0.28**					
5. Posttest intrinsic motivation	4.71	1.55	–0.01	0.51**	0.30**	0.88**				
6. Pretest creativity (brick)	3.73	2.54	0.02	–0.04	–0.04	0.07	0.08			
7. Posttest creativity (mug)	4.31	2.29	0.17**	0.04	0.08	0.10	0.18**	0.52**		
8. Intrinsic motivation change^a^	0.13	0.74	0.19**	0.17**	0.05	−0.16**	0.32**	0.03	0.18**	
9. Creativity change^a^	0.58	2.39	0.14*	0.08	0.12	0.03	0.10	−0.57**	0.41**	0.14*

*N* = 298^*a*^; *N* = 235^*b*^. Reward was coded 0 = no reward (control) and 1 = reward (all reward conditions). * *p* < 0.05. ** *p* < 0.01.

### Tests of Manipulation Checks

Two ANOVAs, excluding the control condition, revealed that the manipulations had their intended effects on the reward conditions. The clarity manipulation had a strong effect on the clarity check [*F*(1, 234) = 180.15, *p* < 0.001] and a non-significant effect on the fairness check [*F*(1, 234) = 0.35, *p* = 0.55], while the evaluation fairness manipulation had a strong effect on the fairness check [*F*(1, 234) = 21.74, *p* < 0.001] and a non-significant effect on the clarity check [*F*(1, 234) = 1.04, *p* = 0.31]. See Table 2 for means and standard deviations of the manipulations for each condition.

**TABLE 2:** Means and standard deviations by condition.

**Condition**	**Intrinsic motivation (pretest)**	**Intrinsic motivation (posttest)**	**Intrinsic motivation change**	**Creativity-brick (pretest)**	**Creativity-mug (posttest)**	**Creativity change**
Control (no reward; *n* = 63)	4.85 (1.24)	4.71 (1.41)	**–0.14 (0.58)**	3.65 (2.52)	3.58 (2.02)	**–0.07 (2.15)**
Low allocation clarity (*M* = 2.79, *SD* = 1.70), low evaluation fairness (*M* = 4.11, *SD* = 1.56; *n* = 56)	4.27 (1.46)	4.39 (1.73)	**0.12 (0.74)**	3.91 (2.94)	4.46 (2.52)	**0.55 (2.29)**
Low allocation clarity (*M* = 3.48, *SD* = 1.74), high evaluation fairness (*M* = 5.10, *SD* = 1.39; *n* = 58)	4.50 (1.57)	4.72 (1.62)	**0.22 (0.81)**	3.74 (2.53)	4.8 (2.38)	**1.06 (2.72)**
High allocation clarity (*M* = 5.83, *SD* = 1.36), low evaluation fairness (*M* = 4.35, *SD* = 1.60; *n* = 62)	4.55 (1.54)	4.63 (1.55)	**0.08 (0.70)**	4.17 (2.44)	4.46 (2.52)	**0.29 (2.45)**
High allocation clarity (*M* = 5.81, *SD* = 1.21), high evaluation fairness (*M* = 5.12, *SD* = 1.14; *n* = 59)	4.67 (1.59)	5.07 (1.41)	**0.40 (0.77)**	3.19 (2.22)	4.32 (1.83)	**1.13 (2.15)**

*Standard deviations are in parentheses under each average. Change variables are in bold.*

### Tests of Reward Conditions Versus Control Condition

A visual inspection of the changes in intrinsic motivation and creativity showed increases for each of the reward conditions in contrast to participants in the control group who experienced decreases in intrinsic motivation and creativity (Table 2).

Next, one-way multivariate analysis of variance (MANOVA) was used four times to compare the control condition in intrinsic motivation change (*M* = -0.14, *SD* = 0.58) and creativity change (*M* = -0.07, *SD* = 2.15) to each of the reward conditions. Significant multivariate effects were followed by univariate *F*-tests, and they revealed the following: ***Low allocation clarity/low evaluation fairness*** showed a non-significant difference on combined dependent variables [*F*(2, 116) = 2.94, *p* = 0.057, Wilk’s Lambda = 0.95, and partial eta squared = 0.05]. ***Low allocation clarity/high evaluation fairness*** had a significant difference on combined dependent variables [*F*(2, 118) = 6.33, *p* = 0.002, Wilk’s Lambda = 0.90, and partial eta squared = 0.10]. Both intrinsic motivation change [*M* = 0.22, *SD* = 0.81; *F*(1, 119) = 8.11, *p* = 0.005, and partial eta squared = 0.06] and creativity change [*M* = 1.06, *SD* = 2.72; *F*(1, 119) = 6.52, *p* = 0.012, and partial eta squared = 0.05] were significant when considered separately. ***High allocation clarity/low evaluation fairness*** had a non-significant difference on combined dependent variables [*F*(2, 122) = 2.11, *p* = 0.13, Wilk’s Lambda = 0.97, and partial eta squared = 0.03]. ***High allocation clarity/high evaluation fairness*** condition had a significant difference on the combined dependent variables [*F*(2, 119) = 13.55, *p* < 0.001, Wilk’s Lambda = 0.82, and partial eta squared = 0.19]. Both intrinsic motivation change [*M* = 0.40, *SD* = *0.77*; *F*(1, 120) = 19.50, *p* < 0.001, and partial eta squared = 0.14] and creativity change [*M* = 1.13, *SD* = 2.15; *F*(1, 120) = 9.47, *p* = 0.003, and partial eta squared = 0.07] were significant when considered separately. Table 3 displays the results for each reward condition when compared with the control condition (all univariate *F* values are reported, including those with non-significant multivariate tests).

**TABLE 3:** Multivariate and univariate analyses of variance *F* ratios for changes in intrinsic motivation and creativity as a function of reward condition versus control condition.

				**Univariate**	
	**Multivariate**			**Intrinsic motivation change**	**Creativity change**
**Condition**	***df***	***F***	***df***	***F***	***F***
Low allocation clarity, low evaluation fairness	*F*(2, 116)	2.94	*F*(1, 117)	4.56*	2.36
Low allocation clarity, high evaluation fairness	*F*(2, 118)	6.33**	*F*(1, 119)	8.11**	6.52*
High allocation clarity, low evaluation fairness	*F*(2, 122)	2.11	*F*(1, 123)	3.63	4.09
High allocation clarity, high evaluation fairness	*F*(2, 119)	13.55***	*F*(1, 120)	19.50***	9.47**

*F* ratios are Wilk’s approximation of *F*. * *p* < 0.05. ** *p* < 0.01. *** *p* < 0.001.

Thus, the two reward conditions with high evaluation fairness had significant increases in both intrinsic motivation and creativity compared to the control condition. On the other hand, the high reward allocation clarity conditions did not exhibit significant changes in intrinsic motivation and creativity.

### Mediation Tests

Finally, to better understand how fairness and timeliness related to both intrinsic motivation and creativity, I employed two mediation tests with the PROCESS (v3) macro (Hayes, 2017) using a percentile bootstrap with 5,000 samples. First, controlling for reward allocation clarity, I tested whether intrinsic motivation mediated the effect of evaluation fairness on creativity. Results indicated that procedural fairness was a significant predictor of intrinsic motivation, *B* = 0.50, *SE* = 0.07, *p* < *0.001*, and that intrinsic motivation was a significant predictor of creativity, *B* = 0.35, *SE* = 0.11, *p* < 0.01. There was no significant direct effect of evaluation fairness on creativity, but the indirect effect was significant, *B* = 0.17, *SE* = 0.06, and 95% CI = 0.059,0.300. These results indicate that intrinsic motivation mediates the relationship between procedural fairness and creativity.

I also tested whether intrinsic motivation mediated the relationship between allocation clarity and creativity after controlling for fairness. Allocation clarity was not a significant predictor of intrinsic motivation, *B* = 0.08, *SE* = 0.05, *p* = *0.10*, there was no significant direct effect on creativity, and the indirect effect was also not significant, *B* = 0.03, *SE* = 0.02, 95% CI = -0.008,0.086. These results did not support a relationship between allocation clarity and creativity with intrinsic motivation as a mediator.

Ultimately, the mediation tests reveal why the conditions with high procedural fairness increased in intrinsic motivation and creativity. This was due to an indirect relationship between procedural fairness and creativity mediated by intrinsic motivation.

## Discussion

This is one of the first studies to examine the effects of procedural justice elements specifically in relation to the reward, intrinsic motivation, and creativity relationship. The findings provide support for the ability of creativity-contingent rewards to positively influence intrinsic motivation and creativity if the procedures used in determining reward recipients are fair. Common sense would dictate that fair procedures should be in place for decision-related processes, yet managers do not always apply the principles of justice (Folger and Skarlicki, 2001); this study provides a valuable reminder of the importance of applying fair procedures to reward evaluation.

Creativity-contingent rewards led to significant increases in intrinsic motivation and creativity in the high reward evaluation fairness conditions, but not in the high reward allocation clarity conditions. Therefore, the results show that that there are exceptions to Byron and Khazanchi’s (2012) conclusion that creativity-contingent rewards increase creativity. This study showed that this was the case only when rewards were evaluated fairly, thereby increasing intrinsic motivation. Besides signaling that creativity is valued, creativity-contingent rewards can increase creativity if they positively impact intrinsic motivation, since changes in levels of intrinsic motivation are likely to impact changes in creativity. In support of this relationship, the mediation tests gave evidence of the indirect relationship between evaluation fairness and creativity through intrinsic motivation.

High reward evaluation fairness of creativity-contingent rewards led to higher increases in intrinsic motivation and creativity compared to the control (no reward) condition and to the reward conditions with low reward evaluation fairness. Two primary mechanisms were suggested for this; one relating to positive affect and the other relating to the fulfillment of basic psychological needs as posited by SDT. In addition, the two mechanisms may share a causal relationship such that need fulfillment precedes positive affect (Sheldon et al., 2001), which leads to intrinsic motivation and creativity. Previous research has shown that positive affect mediates the effects of procedural justice on performance (Colquitt et al., 2013), but fairness perceptions may also influence intrinsic motivation and creativity directly through need satisfaction (Aryee et al., 2015). This may be because fair processes better enable individuals to predict outcomes, leading to a sense of control (i.e., autonomy), they help individuals to attribute favorable outcomes to their own doing, leading to positive self-regard (i.e., competence), and they assist with bringing individuals closer together, leading to a sense of belonging (i.e., relatedness; Cropanzano et al., 2001).

Surprisingly, high reward allocation clarity of creativity-contingent rewards did not lead to higher increases in intrinsic motivation and creativity. This was unexpected, and it demonstrates that elements of procedural justice can vary in their influence. Also, it is not the first time that an element of justice has shown non-significant effects on motivation and performance (Colquitt et al., 2006; Roberson and Stewart, 2006; Zapata-Phelan et al., 2009). Ultimately, in the context of this study, reward evaluation fairness was more influential to changes in intrinsic motivation and creativity than the timeliness of reward allocation.

### Practical Implications

The inspiration for applying justice to the reward–motivation–creativity debate came from R&D employees and patent managers in a large multinational organization. For them, the clarity of reward allocation and fairness of patent remuneration procedures were critical factors in how they perceived the overall fairness of the process used to determine and allocate patent rewards, and that these facets of justice in relation to rewards could potentially influence intrinsic motivation and creativity. Thus, the behavioral experiment stems from experiences in the field, and the results are therefore of clear practical relevance.

Pay for performance is common in organizations (Rynes et al., 2005), and rewards are sometimes dependent on creativity-related performance in organizations (Burroughs et al., 2011). However, the deleterious effects of rewards on intrinsic motivation and creativity have been observed in scholarly research, and those findings have even been popularized by the best-selling book, *Drive* (2009), by Pink, 2009. Contrary to providing evidence of the negative effects of rewards on intrinsic motivation and creativity, this study provides evidence of their positive impact under fair procedures.

Although reward allocation clarity was not influential in this study, it is certainly possible that in practical settings, the results could be different, especially over longer periods of time, or with repeated experiences of unclear and untimely reward allocation. Ultimately, the consequences of rewards are complicated by the myriad possible conditions and contexts in which they can be offered and their complex effects require a nuanced approach (Sansone and Harackiewicz, 1998; Byron and Khazanchi, 2012). This study shows the beneficial potential of rewards under specific conditions, and it is therefore advisable to take a careful approach to providing rewards.

### Limitations and Future Research

There are several limitations to this study. Although this study was inspired by practice-relevant issues, caution should be applied in generalizing or directly transferring the findings to the workplace. The laboratory allowed for isolation of the procedural justice aspects of reward allocation clarity and reward evaluation fairness. The laboratory also allowed for measurement of intrinsic motivation and creativity at two points in time, while permitting for both a control condition and four reward conditions among adults who were already working toward a monetary reward. Although participants were not asked whether they were employed; recent studies using samples from MTurk have shown participant employment rates of around 80% (Ganegoda et al., 2016), which may indicate that the results are more generalizable to the general working population than other laboratory studies that use children or college students as participants. Finally, the usage of a laboratory study allowed for random assignment, which can eliminate alternative explanations and thus strengthens internal validity.

There are limitations with the manipulations and how intrinsic motivation and creativity were measured. First, the manipulations may not have been as precise as desired. The clarity concept likely did not fully address the concept of clarity while the fairness concept may have also been confounded with importance. Second, I only used self-report measures for intrinsic motivation, and I did not include free-choice measures. Although multiple studies have only used self-report measures, it has been suggested that both should be included in research when possible (Deci et al., 1999). However, free-choice measures would have been difficult to assess remotely, and they may not always represent intrinsic motivation anyway (Ryan et al., 1991), especially in relation to extrinsic rewards in work settings (Wiersma, 1992; Gerhart and Fang, 2014). Third, I measured creativity with a divergent thinking task. Although this is a common way to measure creativity, this type of task more accurately measures creative potential (Runco, 2010), or the capacity for idea generation (Reiter-Palmon et al., 2019). Moreover, there are many considerations to be made when using divergent thinking tests (e.g., instructions, time, and scoring, etc.), and in line with Reiter-Palmon et al. (2019), I have been careful to report a detailed account of the tasks employed in this study.

Another limitation includes the lack of measurement of the underlying mechanisms for the relationships between procedural justice, intrinsic motivation, and creativity. Neither affect nor the basic needs of autonomy, competence, and relatedness were measured, even though they were proposed as mechanisms leading to changes in intrinsic motivation and creativity. Previous research has shown a relationship between justice and positive affect (Colquitt et al., 2013), between need satisfaction and autonomous motivation (Gagné et al., 2015; Thibault Landry et al., 2017), and between justice, the satisfaction of needs, and intrinsic motivation (Aryee et al., 2015; Olafsen et al., 2015). Thus, future research on creativity-contingent rewards could include need satisfaction and/or affect as mediators between procedural justice and intrinsic motivation.

Finally, this study only tested short-term effects of creativity-contingent rewards. Although short-term increases of intrinsic motivation and creativity could be beneficial to organizations, it is not clear from this study whether long-term increases in intrinsic motivation and creativity can be gained by using rewards, even those with fair assessment procedures. The effects of reward allocation clarity might become more apparent over long time periods, while unjust reward evaluation and allocation procedures could have potentially unfavorable consequences for organizations and their employees, including decreased innovation and job satisfaction. Future research—especially organizational field studies—could address these issues.

## Data Availability Statement

The datasets generated for this study are available on request to the corresponding author.

## Ethics Statement

Ethical review and approval was not required for the study on human participants in accordance with the local legislation and institutional requirements. Written informed consent for participation was not required for this study in accordance with the national legislation and the institutional requirements.

## Author Contributions

The author confirms being the sole contributor of this work and has approved it for publication.

## Conflict of Interest

The authors declare that the research was conducted in the absence of any commercial or financial relationships that could be construed as a potential conflict of interest.
